# Drug-interaction between paclitaxel and goshajinkigan extract and its constituents

**DOI:** 10.1007/s11418-021-01552-8

**Published:** 2021-07-25

**Authors:** Akiko Nakayama, Kazuaki Tsuchiya, Lingyu Xu, Takashi Matsumoto, Toshiaki Makino

**Affiliations:** 1 Tsumura Advanced Technology Research Laboratories, Kampo Research and Development Division, Tsumura & Co., Ibaraki, 300-1192 Japan; 2 Department of Pharmacognosy, Graduate School of Pharmaceutical Sciences, Nagoya City University, Nagoya, Japan

**Keywords:** Goshajinkigan, Paclitaxel, Drug-drug interaction, Metabolism, CYP2C8, CYP3A4

## Abstract

Paclitaxel, a standard chemotherapeutic agent for several types of cancer, including ovarian, breast, and non-small-cell lung cancer, causes peripheral neuropathy as an adverse effect in 60–70% of the patients. The utility of combination therapy with paclitaxel and goshajinkigan, a traditional Japanese Kampo medicine, in managing paclitaxel-induced neuropathy during chemotherapy has been explored. Paclitaxel is predominantly metabolized in the liver by cytochrome P450 (CYP) 2C8 to produce 6α-hydroxypaclitaxel and by CYP3A4 to produce 3′-p-hydroxypaclitaxel. In this study, we evaluated the inhibitory or inducing effects of goshajinkigan extract (GJG) and its representative and bioavailable constituents, geniposidic acid, plantagoguanidinic acid, paeoniflorin, catalpol, loganin, and neoline, on the metabolism of paclitaxel via CYP2C8 and CYP3A4 using pooled human liver microsomes and cultured human cryopreserved hepatocytes to provide the drug information about the pharmacokinetic interaction of this combination therapy. GJG significantly inhibited the production of 3’-p-hydroxypaclitaxel and 6α-hydroxypaclitaxel in vitro in a concentration-dependent manner. The half maximal inhibitory concentration (IC_50_) values of GJG were 4.5 and 7.8 mg/ml, respectively, for 3′-p-hydroxypaclitaxel and 6α-hydroxypaclitaxel productions. Neoline inhibited the production of 3′-p-hydroxypaclitaxel at 50 μM, but not at lower concentrations. Apart from neoline, other GJG constituents (at concentrations up to 50 or 10 μM of all test substances) did not exhibit inhibitory or inducing effects. Since GJG showed the inhibitory effect on the metabolism of paclitaxel at much higher concentrations than those used clinically, it can be concluded that GJG product does not exhibit any pharmacokinetic interaction with paclitaxel in clinical practice.

**Graphic abstract:**

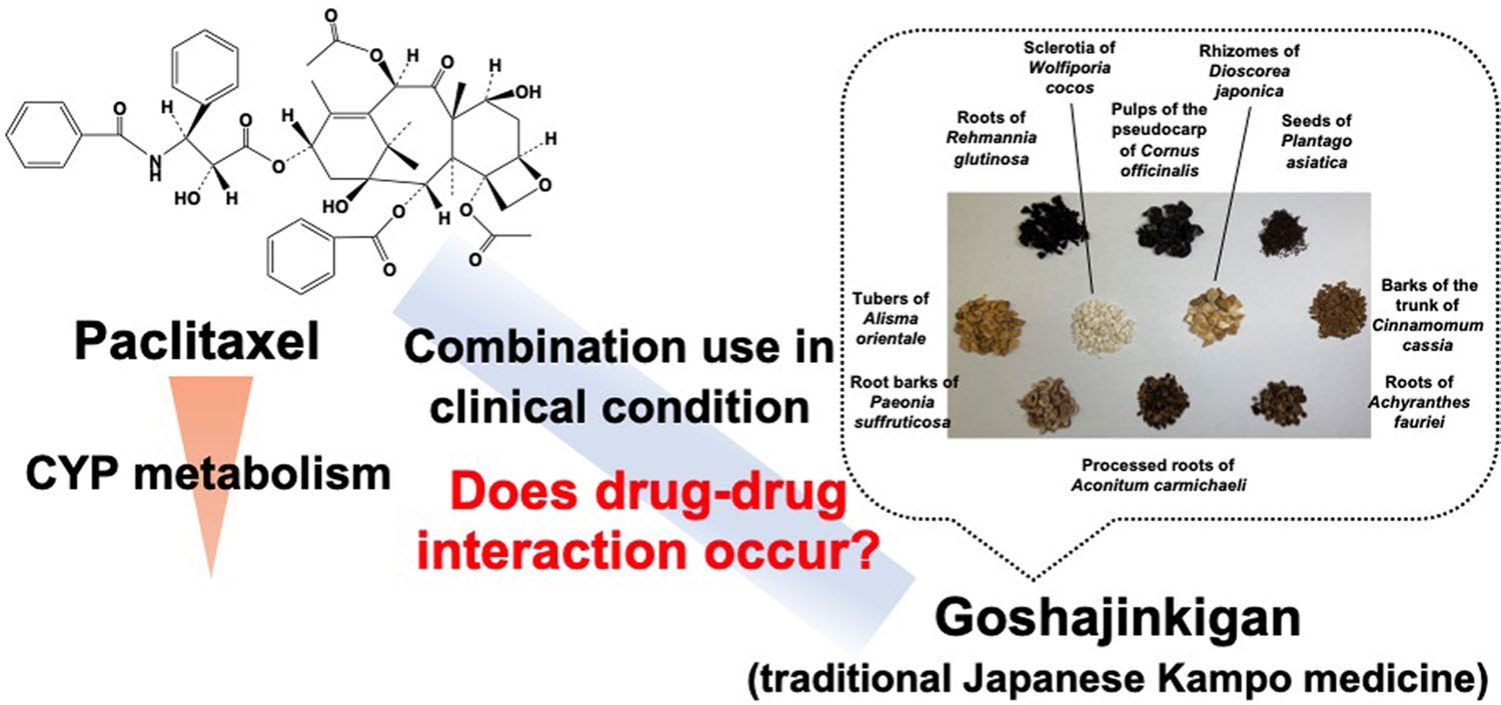

## Introduction

Paclitaxel, a tricyclic diterpenoid compound derived from the bark and needles of *Taxus brevifolia*, is a standard chemotherapeutic agent for several types of cancer, including melanoma, ovarian, breast, and lung, bladder, and prostate cancers. The National Comprehensive Cancer Network guidelines has recommended its use in non-small-cell lung cancer in patients [1]. Similar to other chemotherapeutic agents, paclitaxel causes peripheral neuropathy as a side effect. The incidence rate is reported to be 60–70% among chemotherapy patients, and this side effect is a major reason for early termination of paclitaxel chemotherapy [2]. To manage paclitaxel-induced neuropathy during chemotherapy, combination therapy with paclitaxel and Japanese traditional Kampo medicines has been explored. A clinical study previously reported that about 20% of the patients who have neuropathy under the paclitaxel chemotherapy was prevented by the combination therapy with goshajinkigan extract (GJG) product [3]. Goshajinkigan is a formula of Kampo medicine composed of ten crude drugs including the dried root of *Rehmannia glutinosa* (Rehmannia root), the dried root of *Achyranthes fauriei* (Achyranthes root), the dried pulp of the pseudocarp of *Cornus officinalis* (Cornus fruit), the dried rhizome of *Dioscorea japonica* (Dioscorea rhizome), the dried seeds of *Plantago asiatica* (Plantago seed), the dried tubers of *Alisma orientale* (Alisma tuber), the dried sclerotia of *Wolfiporia cocos* (Poria sclerotium), the dried root bark of *Paeonia suffruticosa* (Moutan bark), the dried bark of the trunk of *Cinnamomum cassia* (Cinnamon bark), and the dried roots of *Aconitum carmichaeli* after autoclaved (processed Aconite root). Indications of ethical GJG product that has been approved by the Medicinal Regulatory Agency in Japan are to relieve leg pain, lower back pain, numbness, blurred vision in the elderly, pruritus, dysuria, frequent urination and edema [4, 5]. In animal experiments, it is revealed that GJG prevents paclitaxel-induced neuropathy without impairing anti-tumor activity of paclitaxel [6], and that the extracts of Plantago seed and processed Aconite root, and their ingredients aucubin and neoline, respectively, exhibited the preventive effects on paclitaxel-induced neuropathy [7, 8].

The use of traditional herbal medicines continues to expand rapidly worldwide, the information on drug interactions between herbal medicines and chemical drugs is therefore needed [9]. The most well-known drug interaction between herbal medicines and chemical drugs is the case of grapefruit juice [10], wherein furanocoumarins (the active constituents) inhibit intestinal cytochrome P450 (CYP) 3A4 and consequently increase the bioavailability of its substrates, such as felodipine, cyclosporine and carbamazepine [11]. St. John's wort and its constituent hyperforin induce the expression of CYP2B6, CYP2C9, CYP3A4, and CYP3A5, resulting in reduced blood concentrations of the substrates of these CYPs [12]. The extracts of tokishakuyakusan, kamishoyosan, and keishibukuryogan, the three major Kampo formulations frequently used in gynecology, significantly inhibited CYP3A4 activity at higher concentrations in vitro. However, at regular doses, Kampo formulations exhibit negligible inhibitory effects, and therefore do not cause significant interactions with CYP3A4 substrates [13].

Paclitaxel is metabolized by two different CYPs: CYP2C8 catalyzes the 6-hydroxylation at the taxane ring of paclitaxel to produce 6α-hydroxypaclitaxel, and CYP3A4 oxidizes the tert-butyl group of the lateral chain in C13 of docetaxel to produce 3'-p-hydroxypaclitaxel [14]. The liver is the major metabolic organ, and the fraction of the dose metabolized was estimated to be 0.963 based on paclitaxel human absorption, distribution, metabolism, and excretion data [15, 16]. The bioavailability of paclitaxel increases when hepatic metabolism by CYP enzymes is inhibited by concomitant drug(s). Indeed, a clinical case of drug-drug interaction of paclitaxel via CYP inhibition has already been confirmed; when pazopanib, an inhibitor of CYP2C8 and CYP3A4, reduced the clearance of paclitaxel and enhanced the maximum concentration [17]. Thus, understanding the inhibitory or inducing effects of Kampo medicines on CYPs is very important in preventing serious drug interactions when used in combination with paclitaxel.

In this study, we evaluated the inhibitory and inducing effects of GJG and its representative and bioavailable constituents (geniposidic acid, plantagoguanidinic acid, paeoniflorin, catalpol, loganin, and neoline) on the metabolism of paclitaxel in vitro. These six compounds were chosen since we detected these compounds at comparatively high concentrations in human plasma following oral administration of GJG (in submitting). We attempted to provide useful drug information about combination therapy with GJG product and paclitaxel to prevent peripheral neuropathy during paclitaxel chemotherapy.

## Materials and methods

### Materials

Paclitaxel and rifampicin were purchased from Nippon Kayaku (Tokyo, Japan) for the experiments on cytochrome inhibition, and from Fujifilm Wako Pure Chemical Industries (Osaka, Japan) for the experiments on cytochrome induction. 6α-Hydroxypaclitaxel and 3′-p-hydroxypaclitaxel were purchased from Cayman Chemical (Ann Arbor, MI, USA) and ChromaDex (Santa Ana, CA, USA), respectively. Geniposidic acid was purchased from ChemFaces (Wuhan, China). GJG graded according to the quality standards specified in the Japanese Pharmacopoeia 17th Edition (JP17) [18], plantagoguanidinic acid, paeoniflorin, catalpol, loganin, and neoline were supplied by Tsumura & Co. (Tokyo, Japan). GJG was the dried extract of the following prescription: Rehmannia root (5 g), Cornus fruit (3 g), Dioscorea rhizome (3 g), Alisma tuber (3 g), Poria sclerotium (3 g), Moutan bark (3 g), Cinnamon bark (1 g), powdered processed Aconite root (1 g), Achyranthes root (3 g), and Plantago seed (3 g) [4]. All crude drugs were also graded according to the quality standards specified in JP17, and the fingerprint pattern of GJG was shown in our previous study [19]. Pooled human liver microsomes (mixed gender) were purchased from Sekisui XenoTech, LLC. (Kansas City, KS, USA). *n*-Butyl *p*-hydroxybenzoate (BHB), quercetin, and niflumic acid were purchased from Nacalai Tesque (Kyoto, Japan), Tokyo Chemical Industry (Tokyo, Japan), and Sigma-Aldrich (St. Louis, MO, USA), respectively.

### Assay of inhibition of paclitaxel metabolism by GJG and its constituents in human liver microsome fraction

We used the modified methods reported previously [13]. In brief, 10 µl of GJG sample solution, 76 µl of 20 µM paclitaxel in 0.15 M phosphate buffer (pH 7.4) containing 0.13 M EDTA, and 4 µl of human liver microsomes (3 mg/ml diluted with phosphate buffer) were mixed and incubated at 37 °C for 5 min. Then, 10 µl of 10 mM β-NADPH (Oriental Yeast Co., Ltd., Tokyo, Japan) was added, mixed, and incubated at 37 °C for 140 min. The reaction was stopped by adding 100 µl of ethanol containing 40 µM BHB as an internal standard into the reactant and mixing vigorously. After centrifugation (15,000 × *g*, 7 min), 10 µl of the supernatant was analyzed using HPLC–MS/MS (Quattro Premier XE, Waters, Milford, MA, USA). The mass spectrometer used an electrospray ionization source in the positive ion mode with multiple reaction monitoring. The analytical column was an Inertsil ODS-4 (2.1 mm i.d. × 100 mm, 3 µm) (GL Sciences Inc., Tokyo, Japan). The mobile phase was delivered using a linear gradient elution system consisting of 0.5% formic acid (A): acetonitrile containing 0.5% formic acid (B), at a flow rate of 0.2 ml/min, with the following gradient profile: 40% B (0 min), increasing from 40 to 70% (0–6 min), and 70% B (6–8 min). The transitions (precursor to daughter) monitored and retention times were 195.2–139.0 *m/z* [M + H]^+^ for BHB (3.3 min), 892.4–308.4 *m/z* [M + Na]^+^ for 6α-hydroxypaclitaxel (4.2 min), 892.4–324.0 *m/z* [M + Na]^+^ for 3′-p-hydroxypaclitaxel. The linear regression of the concentration range of 1.00–125 nM of paclitaxel, 6α-hydroxypaclitaxel, and 3′-p-hydroxypaclitaxel by the peak-area ratio of these compounds to BHB was calibrated using the least-squares method (*r*^*2*^ = 0.99). Data were expressed as relative activity (%), defined as the ratio of the content of 6α-hydroxypaclitaxel or 3′-p-hydroxypaclitaxel incubated with the sample to that incubated without the sample. The half-maximal inhibitory concentration (IC_50_) was calculated from the least square regression line made from 3 points that crossed 50% of the control logarithmic concentration values.

### Culture of human cryopreserved hepatocytes

Plateable and interaction-qualified cryopreserved human hepatocytes (Lonza, Walkersville, MD, USA) were thawed in a hepatocyte thawing medium (Lonza) and then seeded at 5 × 10^4^ cells per well in collagen-coated 96-well plates (Corning, Corning, NY, USA) with hepatocyte plating medium (Lonza). After 1 h of incubation with 5% CO_2_ under saturated humidity at 37 °C, the medium was replaced with fresh plating medium. After 4 h of seeding, the media were replaced with maintenance medium (Lonza) containing Matrigel (0.3 mg/ml).

### Assay of induction of paclitaxel metabolism by GJG and its constituents in human cryopreserved hepatocytes

The CYP induction of GJG and its constituents on paclitaxel metabolism were studied using two methods: the evaluation of metabolic activities by analyzing the amounts of 6α-hydroxypaclitaxel and 3′-p-hydroxypaclitaxel produced; and the measurements of mRNA levels.

After removing the medium containing Matrigel from the cells, the cells were treated with each test compound prepared in a maintenance medium (1, 3, and 10 µM). Rifampicin, a well-known CYP2C8 and CYP3A4 inducer, was used as a positive control and treated under the same conditions. The cells were divided into two groups: one for the evaluation of mRNA levels and the other for the evaluation of metabolic activities of paclitaxel. To obtain the samples to evaluate mRNA levels, the cells were collected 24 h after drug treatment (*n* = 3 per test compound). To obtain the samples to evaluate the metabolic activities of paclitaxel, the maintenance medium containing each compound was replaced with fresh medium containing each compound at the concentration every 24 h over a 72-h treatment period. Following the compound treatment period, the culture medium was replaced with maintenance medium containing paclitaxel (10 µM). The supernatants were collected to measure the production of paclitaxel metabolites after 60 min incubations (*n* = 3 per test compound). The concentration of DMSO in the maintenance medium was 0.1%. A cell viability assay using CellTiter-Glo (Promega, Madison, WI, USA) was used to ensure the absence of cell toxicity during treatment with each compound. The incubation supernatants were mixed with acetonitrile containing niflumic acid (internal standard), and the concentrations of 6α-hydroxypaclitaxel and 3’-p-hydroxypaclitaxel were measured using an LC–MS/MS which differs from that used in the inhibition assay in human liver microsomes: Prominence LC20A system (Shimadzu, Kyoto, Japan) and an LCMS-8060 triple quadrupole mass spectrometer (Shimadzu) were used. The mass spectrometer used an electrospray ionization source in the positive or negative ion mode with multiple reaction monitoring. The analytical column was a Capcellpak C_18_ MGIII-H (2.0 mm i.d. × 50 mm, 3 µm) (Osaka Soda, Osaka, Japan) coupled with a guard column (Opti-Guard Fit ODS, Tokyo Chemical Industry, Tokyo, Japan) at 40 °C. The mobile phase was delivered using a linear gradient elution system, 0.2% formic acid (A): acetonitrile (B), at a flow rate of 0.25 ml/min, with the following gradient profile: 30% B (0–1 min), increasing from 30 to 50% (3–7.5 min). As the analytical system and methods used in the induction assay and the inhibition assay were different, the MS transitions and retention times of paclitaxel metabolites in this induction assay were reexamined. The transitions (precursor to daughter) monitored and retention times were 283.15–245.10 *m/z* [M + H]^+^ for niflumic acid (6.6 min), 914.2–541.15 *m/z* [M + HCOO]^−^ for 6α-hydroxypaclitaxel (5.7 min), 870.33–122.20 *m/z* [M + H]^+^ for 3′-p-hydroxypaclitaxel (6.6 min). The linear regression of the concentration range of 25 pM–10 µM of 6α-hydroxypaclitaxel and 100 pM–10 µM of 3′-p-hydroxypaclitaxel, respectively, by the peak-area ratio of these compounds to niflumic acid was calibrated using the least-squares method (*r*^*2*^ = 0.99).

### RNA isolation and quantitative PCR analysis

RNA isolation and cDNA synthesis were carried out using the Superprep II Cell Lysis & RT Kit (Toyobo, Osaka, Japan) according to the manufacturer’s protocol. Real-time PCR analysis was performed using TaqMan assay probes and TaqMan Fast Advanced Master Mix (Thermo Fisher Scientific, Waltham, MA, USA) with a Quantstudio 7 real-time PCR system (Thermo Fisher Scientific) according to the manufacturer’s protocol. TaqMan assay probes used are Hs00604506_m1 (CYP3A4), Hs02383390_s1 (CYP2C8), and Hs99999905_m1 (glyceraldehyde 3-phosphate dehydrogenase; GAPDH). The relative expression levels of the target genes were normalized to that of the housekeeping gene GAPDH. Data are expressed as fold-changes to the vehicle control.

### Statistical analysis

**Fig. 1:**
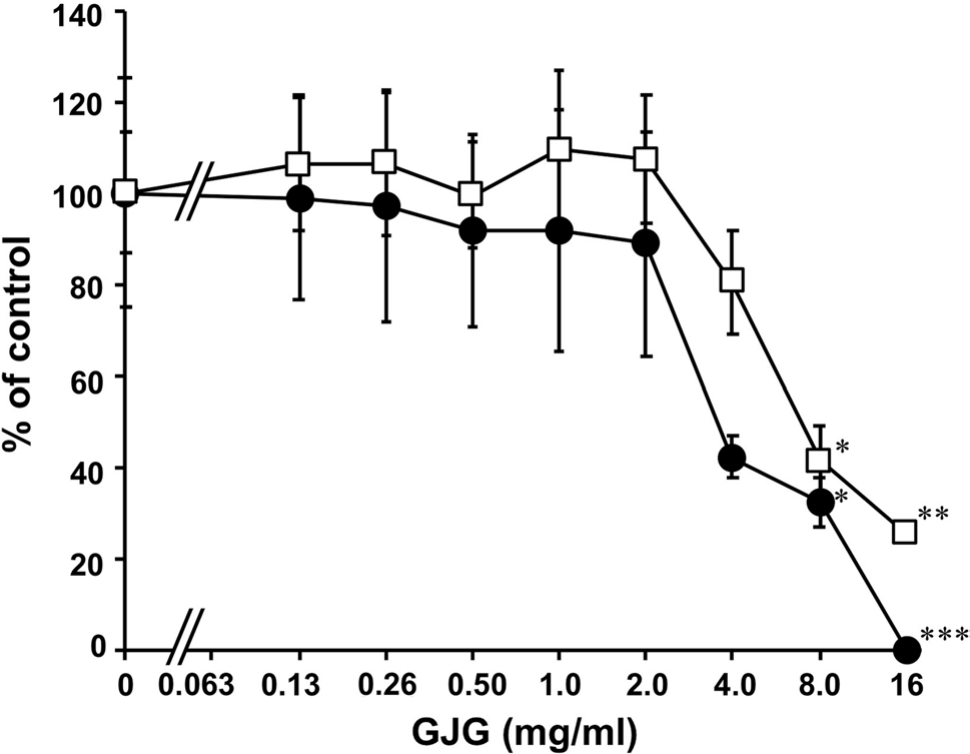
Inhibitory effect of goshajinkigan extract (GJG) on the metabolism of paclitaxel. Paclitaxel and GJG were incubated with human liver microsome fraction and NADPH at 37 ℃ for 140 min, and the reaction was stopped by adding MeOH. The concentrations of 3′-p-hydroxypaclitaxel (closed circle) and 6α-hydroxypaclitaxel (open square) were measured using LC–MS/MS, and the data are shown as % of control (mean ± S.E., *n* = 3). *p < 0.05, **p < 0.01, and ***p < 0.001 vs control group evaluated by Dunett’s *t* test

**Fig. 2:**
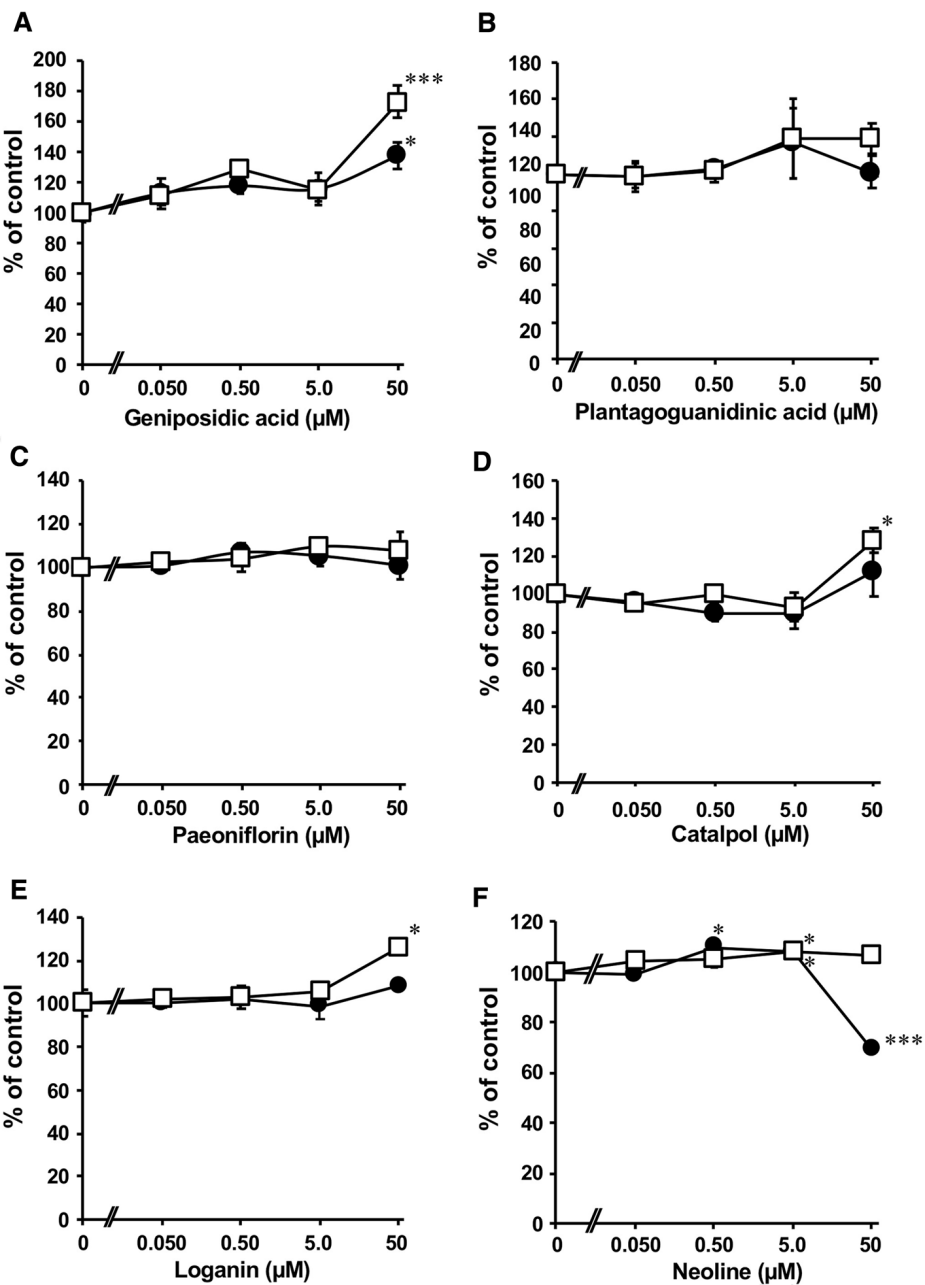
Inhibitory effects representative constituents of goshajinkigan extract (GJG) on the metabolism of paclitaxel. Paclitaxel and test samples were incubated with human liver microsome fraction and NADPH at 37 ℃ for 140 min, and the reaction was stopped by adding MeOH. The concentrations of 3′-p-hydroxypaclitaxel (closed circles) and 6α-hydroxypaclitaxel (open squares) were measured using LC–MS/MS, and the data are shown as % of control (mean ± S.E., *n* = 3). *p < 0.05 and ***p < 0.001 *vs* control group evaluated by Dunett’s *t* test

**Fig. 3:**
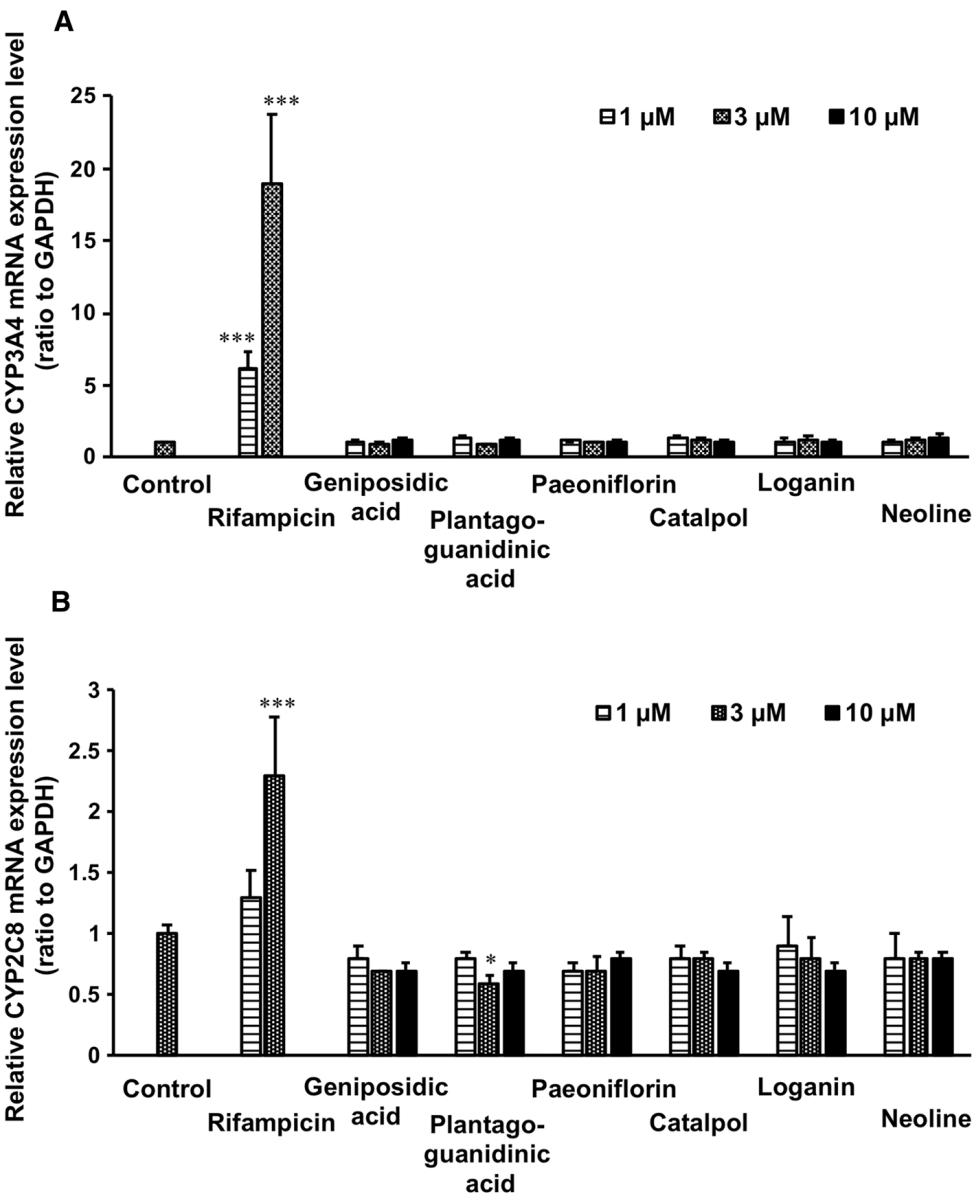
Inducible effects of representative constituents of goshajinkigan extract (GJG) on CYP3A4 and CYP2C8 in human cryopreserved hepatocytes. Human cryopreserved hepatocytes were incubated with test samples for 24 h. Then, total mRNA was collected, and the expression levels of CYP3A4 (**A**) and CYP2C8 (**B**) were evaluated by quantitative PCR analysis. The relative expression level of target mRNA expression to GAPDH, and data are expressed as fold-change to control (mean ± S.D., *n* = 6 in control, *n* = 3 in each chemical). *p < 0.05 and ***p < 0.001 *vs* control group evaluated by Dunett’s *t* test

**Fig. 4:**
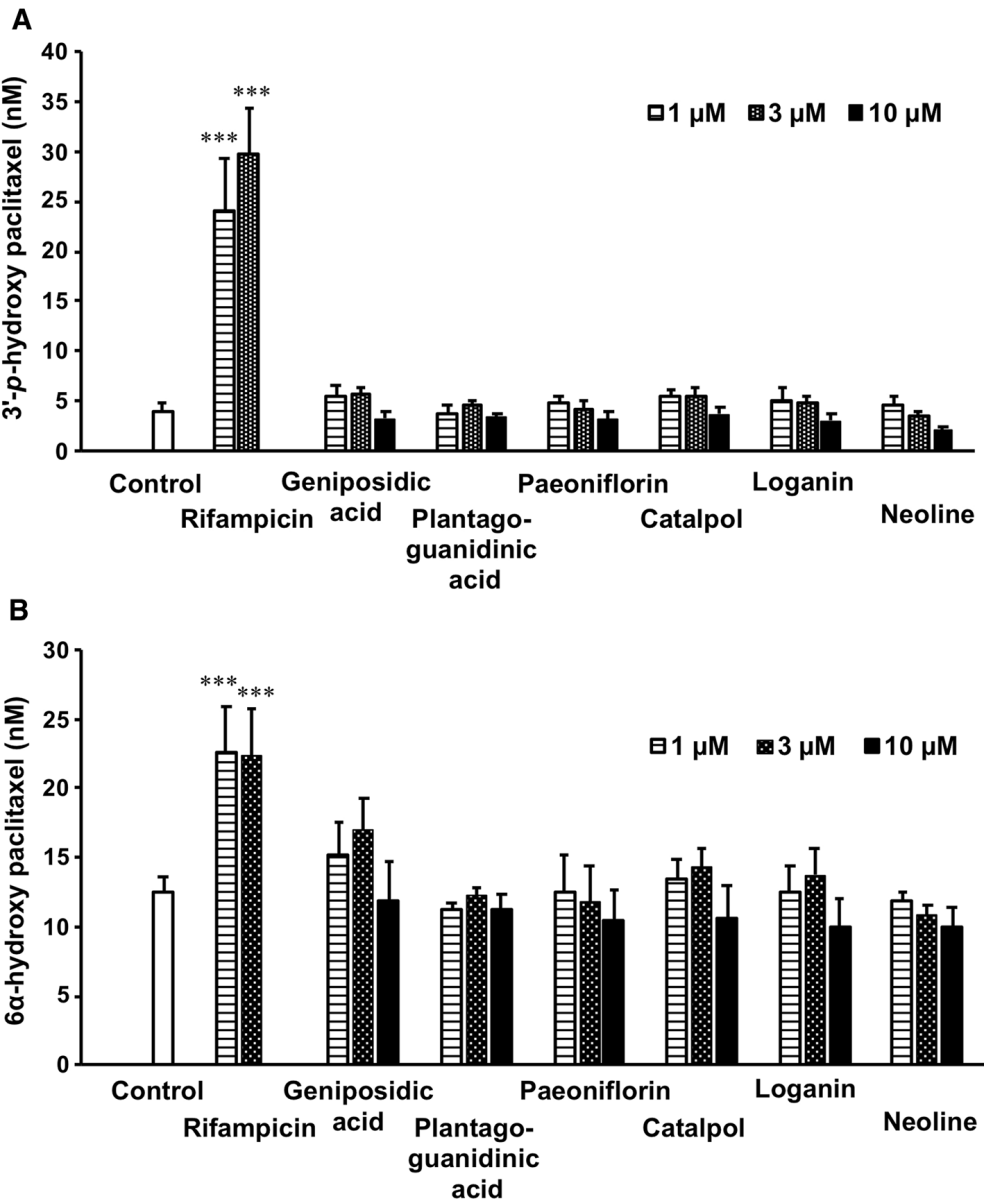
Effect of representative constituents of goshajinkigan extract (GJG) on the metabolism of paclitaxel in human cryopreserved hepatocytes. Human cryopreserved hepatocytes were incubated with test samples for 72 h and then incubated with paclitaxel for 60 min. The concentrations of 3′-p-hydroxypaclitaxel (**A**) and 6α-hydroxypaclitaxel (**B**) were measured using LC–MS/MS, and the data are shown as mean ± S.D. (*n* = 6 in control, *n* = 3 in each chemical). ***p < 0.001 *vs* control group evaluated by Dunnet’s *t*-test

Statistical analyses for each group measured for 6α-hydroxypaclitaxel or 3’-p-hydroxypaclitaxel shown in the graph of Figs. 1 and 2, and all groups shown in the graph of Figs. 3 and 4 were performed using one-way analysis of variance (ANOVA) and Dunnett’s multiple *t*-test using PASW Statistics (version 18, SPSS; IBM, Armonk, NY, USA), respectively. A probability value less than 0.05 was considered statistically significant.

## Results

### Inhibitory effect of GJG on paclitaxel metabolism in vitro

To confirm the reliability of the assay of inhibition of paclitaxel metabolism using human liver microsomes, the inhibitory effect of quercetin, used as a positive control for the inhibition of CYP2C8 [20], was preliminarily investigated. Resultantly, quercetin inhibited the production of 6α-hydroxypaclitaxel by 49% (% of control) at 0.3 µM and 29% at 3 µM. GJG significantly inhibited the production of both 3′p-hydroxypaclitaxel and 6α-hydroxypaclitaxel in vitro in human liver microsomes in a concentration-dependent manner (Fig. 1), and IC_50_ of GJG were 4.5 and 7.8 mg/ml, respectively.

### Inhibitory effects of the constituents of GJG on paclitaxel metabolism in vitro

Figure 2 shows the inhibitory effect of the constituents of GJG on paclitaxel metabolism in vitro by human liver microsomes. Geniposidic acid, catalpol, and loganin significantly induced the production of 6α-hydroxypaclitaxel at 50 µM, and genipodic acid induced the production of 3′-p-hydroxypaclitaxel at 50 µM. Neoline significantly induced the production of both metabolites at 5.0 µM. However, at 50 µM, neoline significantly inhibited the production of 3′-p-hydroxypaclitaxel. Paeoniflorine and plantagoguanidinic acid did not affect paclitaxel metabolism at any concentration, ranging from 0.050 to 50 µM.

### Inducing effects of the constituents of GJG on the CYP3A4 and CYP2C8 mRNA expressions and metabolic activities of paclitaxel in human hepatocytes

Figure 3 shows mRNA expressions of CYP3A4 and CYP2C8 in human cryopreserved hepatocytes treated with the samples. Rifampicin, used as a positive control [21], significantly induced both CYP3A4 and CYP2C8 mRNA expressions in a concentration-dependent manner. Plantagoguanidinic acid at 3 µM significantly reduced CYP2C8 mRNA expression. However, other compounds did not exhibit any inducing effect on CYP3A4 and CYP2C8 mRNA expressions.

In addition, the metabolic activity of paclitaxel by the hepatocytes treated with these compounds was evaluated. Rifampicin significantly induced the production of both 3′-p-hydroxypaclitaxel and 6α-hydroxypaclitaxel by human hepatocytes; however, concentration-dependence was only observed in the production of 3′-p-hydroxypaclitaxel. None of the evaluated constituents of GJG had any effect on the production of both 3′-p-hydroxypaclitaxel and 6α-hydroxypaclitaxel by human hepatocytes at 1–10 µM (Fig. 4).

## Discussion

In the present study, GJG inhibited paclitaxel metabolism in vitro in human liver microsomes in a concentration-dependent manner with IC_50_ valuers of 4.5 mg/ml for 3′-p-hydroxypaclitaxel production and 7.8 mg/ml for 6α-hydroxypaclitaxel production. Since Kampo medicines contain many kinds of compounds including not only plant secondary metabolites but sugars and various ions, these inhibitory effects of GJG with high concentrations might not specific inhibitory effects on CYP3A4 and 2C8. The one-time dosage of GJG (3 divided daily dose) is 1.5 g. When GJG is taken with a cup of water (200 ml), the concentration of GJG is calculated as 7.5 mg/ml, and coincided with the IC_50_ values. Therefore, the inhibitory effects of GJG on the metabolism of orally administered substrates of CYPs might appear in small intestine just after administration of GJG. However, since the results of CYP inhibition in in vitro experiments exhibited excessively high potency [22], in vitro results should be interpreted carefully. Indeed, the drugs can be further diluted by intestinal fluid, and not all constituents cannot be absorbed from intestine into the blood circulation. Based on the in vitro inhibitory activity of Kampo medicines on CYP3A4 described in our previous study [13], it is speculated that the possibility that GJG may inhibit the metabolism of paclitaxel in clinical practice might be quite low by such high IC_50_ values. Since Among the crude drug components of GJG, the extracts of Moutan bark had relatively high inhibitory effects on CYP3A4 in vitro [23], and the extracts of Moutan bark, Alisma tuber, Cinnamon bark, and Poria scletotium inhibited CYP3A4 in human liver microsome fraction with IC_50_ values of 0.10 mg/ml, 0.39 mg/ml, 0.42 mg/ml, and 3.0 mg/ml, respectively [13]. It has been reported that the extracts of Dioscorea rhizome inhibited CYP2C8 in the human liver microsomes with IC_50_ values of 0.24 mg/ml [24]. These crude drugs may contribute to the inhibitory effect of GJG on the metabolism of paclitaxel to 3′-p-hydroxypaclitaxel and 6α-hydroxypaclitaxel. However, the in vitro experiment using crude drug extract is limited because the major tissue where paclitaxel is metabolized is liver, and not all the components of GJG can be absorbed from intestine into the circulation.

Next, we evaluated the inhibitory and the inducing effects of the representative constituents of GJG that can be appeared in the blood circulation after the oral administration of GJG on paclitaxel metabolism. Geniposidic acid and plantagoguanidinic acid are the marker compounds of Plantago seed [25, 26], and these compounds can be absorbed in the unchanged form in rats orally treated with Plantago seed extract [27, 28]. Paeoniflorin and catalpol are the marker compounds of Moutan bark and Rehmannia root, respectively, and these compounds are detected in the blood of rats orally treated with shimotsuto extract [29]. Loganin is the marker compound of Cornus fruit and can be absorbed as the original form in rats orally treated Cornus fruit extract [30]. Neoline is the active compound in processed Aconite root for neuropathic pain and is well absorbed from the rat intestine into the circulation [8, 19]. Except neoline, none of these compounds inhibited paclitaxel metabolism in human liver microsomes at concentrations of 50 µM or less in vitro. Although neoline significantly induced the production of these metabolites at 5 µM, the induction ratios were less than 10% without concentration-dependence. In contrast, plantagoguanidinic acid significantly reduced the mRNA expression of CYP2C8 at 3 µM in human cryopreserved hepatocytes without affecting the production of 6α-hydroxypaclitaxel. Therefore, these results are within the margin of error. Some of these compounds exhibited inhibitory or inducing effects on paclitaxel metabolism in human liver microsomes at 50 µM. When the extract of processed Aconite root (1 g/kg as the original crude drug), which is approximately 50 times the daily dosage in humans, is orally administered to rats, the maximum blood concentration of neoline is approximately 80 nM [8]. Therefore, the concentration at 50 µM is much higher than that achieved in clinical practice. It is speculated that these compounds would not exhibit any effects on paclitaxel metabolism, though these in vitro results are difficult to be translated into clinical practice.

In conclusion, the present study provides drug information about combination therapy with GJG product and paclitaxel, and GJG would not cause pharmacokinetic interactions via CYP3A4 and CYP2C8 when the patients are administered the regular dosage. Future clinical studies of GJG product are required to confirm its safe usage and to prevent drug interactions.

## Data Availability

The data used to support the findings of this study are available from the corresponding author upon request.
