# Correction to: Drug-interaction between paclitaxel and goshajinkigan extract and its constituents

**DOI:** 10.1007/s11418-021-01578-y

**Published:** 2021-10-17

**Authors:** Akiko Nakayama, Kazuaki Tsuchiya, Lingyu Xu, Takashi Matsumoto, Toshiaki Makino

**Affiliations:** 1 Tsumura Advanced Technology Research Laboratories, Kampo Research and Development Division, Tsumura & Co., Ibaraki, 300-1192 Japan; 2 Department of Pharmacognosy, Graduate School of Pharmaceutical Sciences, Nagoya City University, Nagoya, Japan

## Correction to: Journal of Natural Medicines 10.1007/s11418-021-01552-8

The article “Drug-interaction between paclitaxel and goshajinkigan extract and its constituents”, written by Akiko Nakayama, Kazuaki Tsuchiya, Lingyu Xu, Takashi Matsumoto and Toshiaki Makino, was originally published electronically on the publisher’s internet portal on 25 July 2021 without open access. With the author(s)’ decision to opt for Open Choice the copyright of the article changed on 8 October 2021 to © The Author(s) 2021 and the article is forthwith distributed under a Creative Commons Attribution 4.0 International License, which permits use, sharing, adaptation, distribution and reproduction in any medium or format, as long as you give appropriate credit to the original author(s) and the source, provide a link to the Creative Commons licence, and indicate if changes were made. The images or other third party material in this article are included in the article’s Creative Commons licence, unless indicated otherwise in a credit line to the material. If material is not included in the article’s Creative Commons licence and your intended use is not permitted by statutory regulation or exceeds the permitted use, you will need to obtain permission directly from the copyright holder. To view a copy of this licence, visit http://creativecommons.org/licenses/by/4.0.

The original article is updated.

